# Supplementary material for: Motor Cortex Stimulation Reversed Hypernociception, Increased Serotonin in Raphe Neurons, and Caused Inhibition of Spinal Astrocytes in a Parkinson’s Disease Rat Model
Source: Cells. 2021 May 11;10(5):1158. doi: 10.3390/cells10051158 (PMC8150310; doi:10.3390/cells10051158)
Supplement: Supplementary file 1 [file cells-10-01158-s001.zip › cells-1194873-supplementary.pdf]

Supplementary Figure 1. Animal weight and immunohistochemistry analysis.

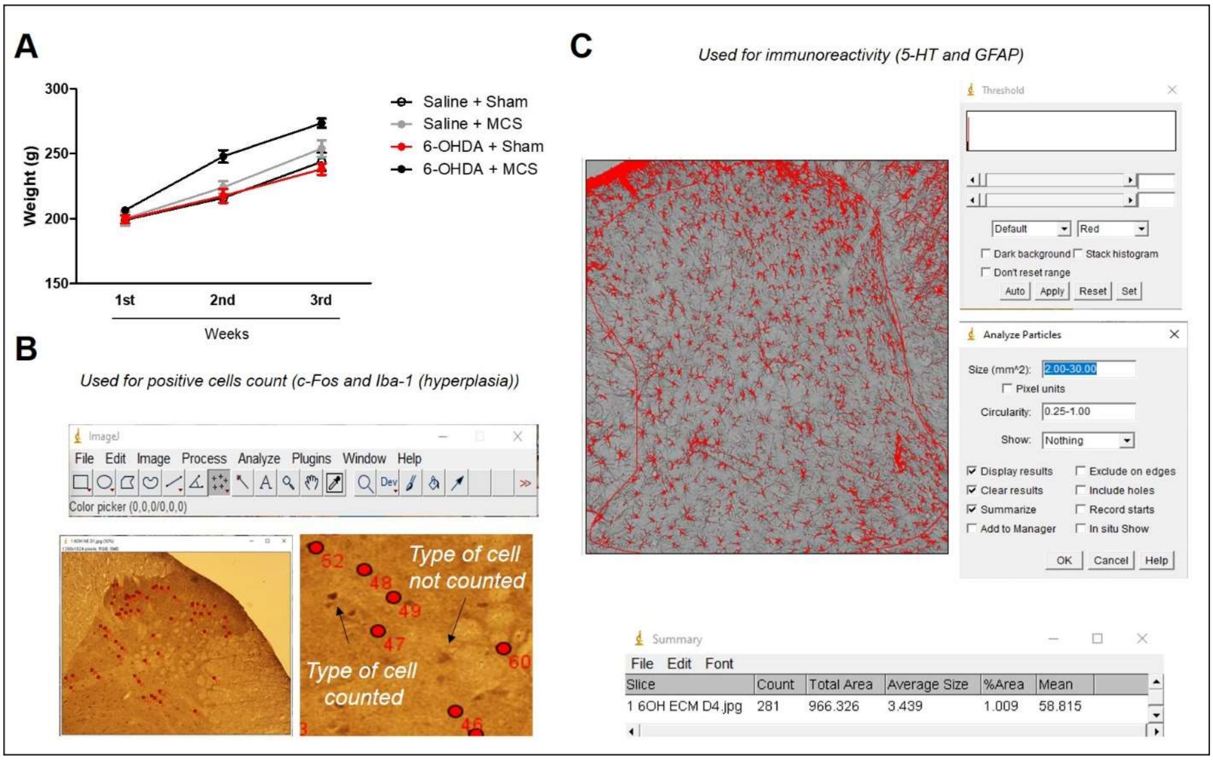

Supplementary Figure 1. Animal weight and immunohistochemistry analysis. Rats were weekly evaluated regarding the weight gain during the experiment design (A). Immunohistochemistry quantification: Cell count scheme used for positive cells analyzes (c-Fos and Iba-1 – hyperplasia) (B). Immunoreactivity (IR) scheme used for quantification of 5-HT-IR and GFAP-IR (C).

Supplementary Figure 2. Photomicrographs of bilateral spinal cord immunohistochemistry.

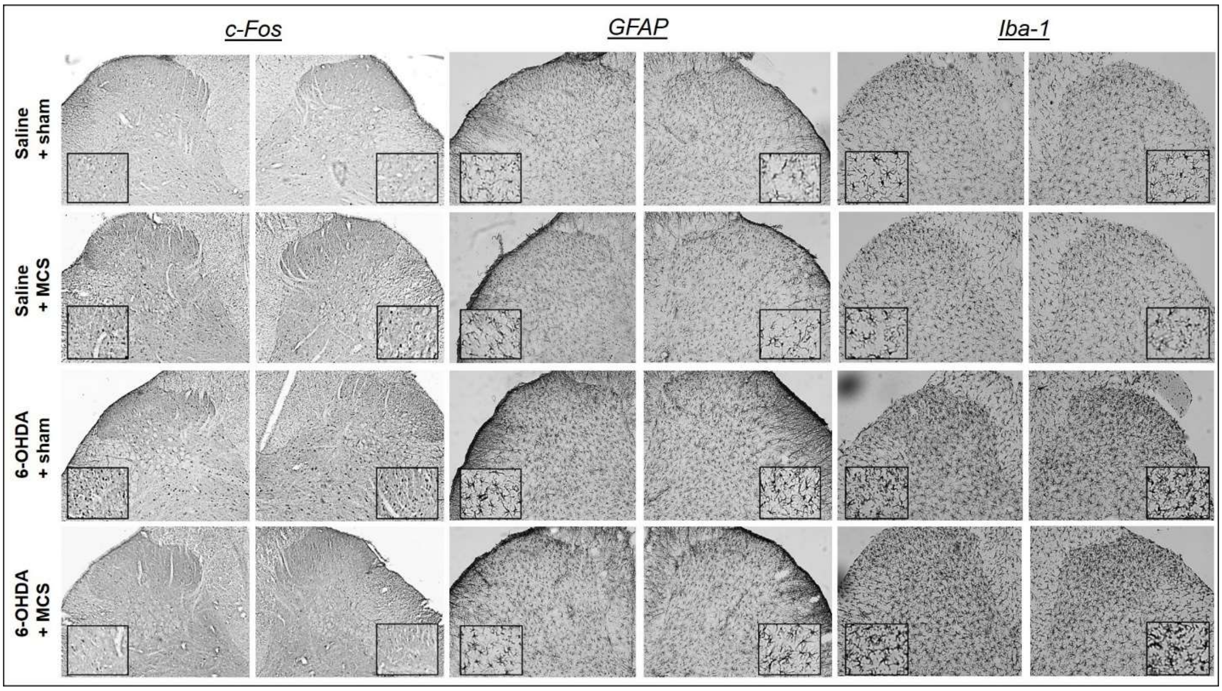

Supplementary Figure 2. Photomicrographs of bilateral spinal cord immunohistochemistry. Neuronal activation pattern and immunoreactivity (IR) for glial cells in the dorsal horn of the spinal cord (DHSC). Photomicrographs of c-Fos-IR, GFAP-IR and Iba-1-IR of rat saline + sham, rat saline + MCS, rat 6-OHDA + sham, and rat 6-OHDA + MCS from left (ipsilateral to the nigrostriatal lesion) and right (contralateral to the nigrostriatal lesion) DHSC. In the small square a higher definition of the labeling of each photomicrograph.
